# Supplementary material for: Correction: A Room with a Viewpoint Revisited: Descriptive Norms and Hotel Guests' Towel Reuse Behavior
Source: PLoS One. 2014 Aug 20;9(8):e106606. doi: 10.1371/journal.pone.0106606 (PMC4139383; doi:10.1371/journal.pone.0106606)
Supplement: File S2 — Republished, corrected article. (PDF) [file pone.0106606.s002.pdf]

A Room with a Viewpoint Revisited: Descriptive Norms and Hotel Guests' Towel Reuse Behavior

Gerd Bohner*, Lena E. Schlüter

University of Bielefeld, Department of Psychology, Bielefeld, Germany

Abstract

Field experiments on descriptive norms as a means to increase hotel guests' towel reuse [1] were replicated and extended. In two hotels in Germany (Study 1: $N = 724$; Study 2: $N = 204$), descriptive norm messages suggesting that 75% of guests had reused their towels, or a standard message appealing to environmental concerns, were placed in guests' bathrooms. Descriptive norm messages varied in terms of proximity of the reference group ("hotel guests" vs. "guests in this room") and temporal proximity (currently vs. two years previous). Reuse of towels was unobtrusively recorded. Results showed that reuse rates were high overall and that both standard and descriptive norm messages increased reuse rates compared to a no-message baseline. However, descriptive norm messages were not more effective than the standard message, and effects of proximity were inconsistent across studies. Discussion addresses cultural and conceptual issues in comparing the present findings with previous ones.

Citation: Bohner G, Schlüter LE (2014) A Room with a Viewpoint Revisited: Descriptive Norms and Hotel Guests' Towel Reuse Behavior. PLoS ONE 9(8): e104086. doi:10.1371/journal.pone.0104086

Editor: Kimmo Eriksson, Mälardalen University, Sweden

Received January 23, 2014; **Accepted** July 9, 2014; **Published** August 1, 2014

Copyright: © 2014 Bohner, Schlüter. This is an open-access article distributed under the terms of the Creative Commons Attribution License, which permits unrestricted use, distribution, and reproduction in any medium, provided the original author and source are credited.

Funding: The authors acknowledge support for the article processing charge by the Deutsche Forschungsgemeinschaft and the Open Access Publication Funds of Bielefeld University Library. The funders had no role in study design, data collection and analysis, decision to publish, or preparation of the manuscript.

Competing Interests: The authors have declared that no competing interests exist.

* Email: gerd.bohner@uni-bielefeld.de

Introduction

Nowadays it is common for travelers to encounter cards placed in hotel bathrooms urging them to reuse their towels. These cards usually appeal to the traveler's environmental consciousness, pointing out the positive effects of towel reuse in terms of saving energy and reducing detergent use. And although the hotel's foremost aim in placing these messages may be saving costs, they may objectively contribute to a cleaner environment. It therefore makes sense to study ways in which the effectiveness of such messages may be improved. Goldstein, Cialdini, and Griskevicius [1] were the first to study the effects of a modified approach: providing descriptive norms. Such norms refer to how most others behave in a given situation and thus inform people about what kind of behavior is likely to be appropriate and effective [2]. Not only were descriptive norm messages (such as "75% of hotel guests have reused their towels") more effective than a standard environmental appeal, but moreover, Goldstein and his colleagues also observed a superior effect of what they called a "provincial norm": When the 75% norm referred to *guests in the same room*, it was more effective than when it referred to all hotel guests [1].

Although studies in a variety of domains have shown that descriptive norms may effectively change behavior [3], there is very little research to date addressing either descriptive norm effects on towel reuse behavior or the more specific effects of provincial versus general norms. Therefore, additional research that aims at replicating those results is called for. We first briefly review the studies by Goldstein and his colleagues [1], along with other available studies that constitute either conceptual replications or approximations of their original design. Then we explain the usefulness of further replication attempts and describe our own studies, which constitute two extended replication experiments that we conducted in hotels in Germany.

Initial Research on Descriptive Norms and Towel Reuse

In their first experiment, Goldstein et al. [1] compared two conditions: a standard environmental message ("HELP SAVE THE ENVIRONMENT. You can show your respect for nature … by reusing your towels during your stay") and a majority descriptive norm ("JOIN YOUR FELLOW GUESTS IN HELPING TO SAVE THE ENVIRONMENT. Almost 75% of guests who are asked to participate in our new resource savings program do help by using their towels more than once …"). Each appeal was followed by instructions to indicate one's intention to reuse (vs. not to reuse) by placing a used towel over the curtain rod or the towel rack (vs. on the floor). Over a period of 80 days, hotel staff recorded the behavior (reuse vs. no reuse) of those guests who stayed at least two nights. Only observations from a guest's first eligible day were analyzed, so each guest participated only once. Results showed a significantly higher reuse rate for the descriptive norm condition (44.1%) than for the standard condition (35.1%).

In their second experiment, Goldstein et al. [1] examined how descriptive norms with different reference groups would affect hotel guests' behavior. Studies have shown that people conform to the norms of groups with whom they share an important social identity [4], or with whom they compare their own group [5]. Taking a different approach, Goldstein and his colleagues [1] hypothesized that "provincial norms," which they defined as "the norms of one's local setting and circumstances" (p. 476) should be more powerful, even if they derive from a social category that is not particularly meaningful to a person's social identity. Thus they compared five conditions: the standard environmental message

and the hotel guest identity norm message (as in their first study) as well as three new normative messages: one referring to guests who stayed in the same room (provincial norm), another referring to fellow citizens, and a final one referring to men and women. All the normative messages stated that about 75% of the reference group had shown the behavior according to a study conducted two years before. As predicted by the authors, the same room condition yielded a higher reuse rate (49.3%) than did the other normative messages combined (42.8%) and the standard message (37.2%). In contrast to this finding, a separate group of participants rated the importance of being a citizen and of being male or female as much more important to their identity than being a hotel guest or a guest in a particular room. Thus, the guests had followed the norm of an immediate, contextual reference group although they probably did not consider this reference group to be relevant to their social identities.

Explanatory Mechanisms and Related Evidence

Although Goldstein et al. [1] acknowledge that the mechanism underlying their observations is not fully clear, they discuss two possibilities. Firstly, people may have learned that local norms are often more diagnostic of appropriate behavior than are more distal norms, and they may overgeneralize this knowledge to settings where the local norm lacks added diagnosticity. Secondly, people may experience a "unit relationship" [6] with others particularly if they share an uncommon characteristic with them. As staying in the same room is more uncommon than staying in the same hotel, the provincial norm effect may be mediated by a stronger unit relationship. (Below we empirically address an additional mechanisms that is not considered by Goldstein et al. [1]: the greater perceived weight and immediacy of one's own contribution in the case of local norms.)

Evidence within other domains appears to be consistent with these conjectures. Research on self-evaluations, for example, has shown that people often rely on low-level, local comparison information while ignoring higher-level, general comparison information, even though they recognize the latter as more diagnostic [7], [8]. In a similar vein, consumer researchers found that purchase decisions may be strongly affected by visible behavior of others in an individual's close environment [9], [10].

Conceptual Replications

Apart from this related evidence, there is one published study, also conducted in the USA, that approximates Goldstein and colleagues' design [11], and one attempt at a direct replication, conducted in Austria and Switzerland [12]. Schultz, Khazian, and Zaleski [11] examined towel reuse in a holiday resort in the USA in a series of studies. In the first of these studies, despite a large sample size (almost 400 observations per condition), presenting a purely descriptive general norm failed to increase reuse rates in comparison to a control condition; only a combination of descriptive and injunctive norms led to a change in behavior. In their third study, Schultz and colleagues [11] compared normative messages with a general (hotel guests) versus provincial norm (guests in same room) as in [1], but – based on their previous results – always combined those descriptive norms with injunctive norms stating that "many … guests have expressed … the importance of conserving energy" (p. 15). In contrast to the original results [1], Schultz and his colleagues [11] observed a descriptively (but not significantly) higher towel reuse rate in the general ("hotel guest") norm condition than in the provincial ("same room") norm condition. Furthermore, only the general norm, but not the provincial norm, was effective in increasing the towel reuse rate compared to a control condition. These apparently diverging results are difficult to interpret, however, because Schultz and colleagues' [11] hotel guest norm condition featured a constant 75% majority, whereas their same room norm condition featured percentages varying between 33% and 92% – reflecting the actual reuse rates that had previously been observed in specific rooms. Nonetheless, their findings point to the possibility that neither the descriptive norm effect per se nor the more specific effect of a provincial norm may be a robust phenomenon.

In a more recent study, Reese, Loew, and Steffgen [12] attempted to directly replicate the original [1] provincial and general norm conditions, as well as a standard environmental appeal condition, in alpine holiday resorts located in Austria and Switzerland. Their dependent variable was the number of towels used per person per day. The results only partly replicated the original findings: Although fewer towels were used in the same room condition ($M = 1.05$) than in the hotel guests condition ($M = 1.63$), overall there was no significant advantage of either of those normative messages over the standard environmental message condition, which descriptively produced a reuse rate falling between the other conditions ($M = 1.32$).

Taken together, the available evidence for descriptive norm effects on towel reuse behavior is mixed. Whereas some studies conducted in the USA point to the possibility that descriptive norms may be effective compared to control conditions [1], [11], but perhaps mainly if they are complemented by an injunctive norm [11], these studies are inconsistent regarding the relative effects of provincial versus general descriptive norms. The one study conducted in Europe [12] replicated an advantage of a purely descriptive provincial norm over a purely descriptive general norm, but failed to show any overall advantage of those descriptive norm conditions in comparisons to a standard environmental message. Its authors speculate that this latter result might reflect a higher baseline of pro-environmental attitudes in Europe as compared to the USA, so that a descriptive norm of 75% combined with a general reference group may be "too irrelevant to elicit higher towel-reuse rates" (p. 99).

The Present Research: An Extended Replication

Replicability of research findings is an important issue for any empirical science [13]. Nonetheless, traditionally, only few replication attempts have been reported in psychology [14]. In recent years, however, the culture of the discipline has been changing toward the active encouragement of replication studies [15], and leading journals that hitherto emphasized the novelty of study ideas now are willing to publish replication attempts, as exemplified by [16]. Replications may support [17] or challenge established assumptions [16], [18], or they may help to specify process assumptions or boundary conditions of a phenomenon [19]. Given the wide impact of the Goldstein et al. paper [1] (between 55 and 69 yearly citations listed in Web of Science for the years 2011 to 2013) and the inconsistencies across studies using similar designs [11], [12], we decided to conduct a direct and extended replication.

In doing so, we took care to use the same dependent variable as did Goldstein and colleagues [1], i.e. whether or not a guest reused his or her towel on the first eligible day. We focused on the comparison of provincial (= same room) norm conditions, general (= hotel guests) norm conditions, and a standard environmental message condition. Furthermore, we extended the design in order to examine a novel mechanism that could be responsible for the provincial norm effect. We reasoned that, in addition to reflecting people's readiness to respond to "the norms of their local setting and circumstances," the superior effect of the same-room

conditions that was observed in previous research [1], [12], might reflect an effect of greater perceived weight or visibility of one's own personal contribution. This would be in line with findings showing that group norms affect public behavior more than private behavior [20], and that people were more motivated to do their best when the perceived number of others acting in the same way was small rather than large [21]. To capture this possible aspect of relative personal visibility, we introduced a temporal manipulation of proximity by stating, depending on condition, that the percentages of people reusing their towels were determined in a study that was completed two years previously – thus directly replicating [1] – or that this percentage was being determined in an ongoing study. If local norms exert a more immediate effect than do more general norms, then norms referring to recent or currently existing social collectives should also exert a more immediate effect than norms referring to social collectives that have existed in the more distal past.

Ethics Statement. Procedures for both Studies 1 and 2 were approved by the Ethics Committee of the Faculty of Psychology and Sports Science at the University of Bielefeld. Because only behavioral traces were to be recorded anonymously and unobtrusively, the ethics committee waived the need for written informed consent from the participants. Labeled datasets from both studies may be obtained by writing to the first author.

Study 1

Method

Participants. Over a five-week period, we collected data on 724 instances of potential towel reuse in all 162 rooms of a four-star hotel located in the center of a mid-sized town in the Northwest of Germany. The vast majority of those observations (714 or 98.6%) were instances of single occupancy, whereas only 10 observations (1.4%) referred to double occupancy. Following the procedures of [1], below we report analyses pertaining to all 724 cases. We also ran additional analyses that included only instances of single occupancy, but as the results did not differ these are not reported in detail.

Materials and Design. For the duration of the study, the hotel's existing towel-reuse message (a standard environmental appeal on a sticker attached to the bathroom mirror) was replaced by one of five messages printed on table tents. These consisted of laminated cardboard, each visible side measuring 11 cm by 14 cm, and were placed in a salient position near the bathroom mirror. The top quarter of each side showed a color photograph depicting some bath towels and the hotel's logo. The bottom three-quarters of each side featured the same message in German and English, respectively. The following messages were used:

- Standard environmental message: "Help to save the environment. Every day we clean a great number of towels, many of them are unused. Please help us to protect the environment. You can join us in this program to help save the environment by reusing your towel during your stay." (This wording was an exact copy of the message the hotel had used previously.)
- Descriptive norm messages: "Join your fellow guests in helping to save the environment. In a study *currently conducted* [*conducted in the fall of 2009*], 75% of the *guests* [*guests who stayed in this room* (#xxx)] participated in our new resource savings program by using their towel more than once. You can join your fellow guests in this program to help save the environment by reusing your towel during your stay."

Text in italics above, outside and within brackets, represents the two levels of the temporal proximity manipulation and the two levels of the general vs. provincial norm manipulation. These were fully crossed to yield four versions of the descriptive norm message. In the provincial norm conditions, "#xxx" was replaced with the actual room number. Exactly replicating [1], each message ended with identical and exact instructions on how to participate or not to participate:

> "If you choose to participate in the program… Please drape the used towel over the towel rack."
> "If you choose not to participate in the program… Please place the towel on the floor or in the shower."

During five weeks, each of the five message versions was used in one of the five floors of the hotel. Each week on Monday, the assignment of a given message to a given floor was changed according to a Latin square design, so that each message was present in each floor for exactly one week.

Procedure. The housekeeping staff was thoroughly instructed how to record reuse rates. To keep procedures as simple as possible, staff members kept track of towels placed on the towel rack on their usual worksheets, which were modified only slightly for the purpose of our study. Each day they ticked separate boxes for each hand towel reused and for each bath towel reused. Although staff members were aware of the different messages being used, they were unaware of any hypotheses. The Executive Housekeeper served as our primary gatekeeper; she monitored proper tracking and confirmed to us that instructions were closely followed.

Test of Manipulations. Because descriptive norms had not been used to influence towel reuse in Germany before, we collected the estimates of a separate group of pilot participants ($N = 64$) to determine if presenting a descriptive norm of 75% would appear credible and effective. A convenience sample of adults was recruited in the area where the hotels of Studies 1 and 2 were located. Pilot participants were between 20 and 68 years old ($M = 28.5$; $SD = 11.4$) and reported having stayed at a hotel during the previous two years between 0 and 22 times ($M = 4.8$; $SD = 5.2$), for a total of 0 to 56 nights ($M = 12.3$; $SD = 10.7$).

Pilot participants completed a questionnaire whose first item asked them to estimate, in an open-ended format, the percentage of people who reuse their towel at least once during a hotel stay of more than one night. Estimates varied between 5% and 100%, with a mean of 46% ($Md = 48\%$; $SD = 28.2\%$). Based on these estimates, presenting a descriptive norm of 75%, as was done in previous studies [1], [11], [12], appeared to be both reasonably credible and potentially effective, being about one standard deviation above people's mean expectancy.

Following [1], pilot participants also rated (1) how much each of our messages would make them think of their identity as an environmentally concerned person, as a hotel guest, and as a guest in a particular hotel room, respectively (response scale from 1, *not at all*, to 5, *very much*), and (2) how important to their identity was being an environmentally concerned person, a hotel guest, and a guest in a particular hotel room (response scale from 1, *not at all important*, to 7, *very important*). Two questionnaire versions were used, one showing the descriptive norm messages in the "current study" version, and one showing them in the "completed study" version. Also, the order in which identities were presented was counterbalanced. (Neither of these variations had any effect on participants' ratings, all $p > .28$.) Pairwise comparisons showed no differences in the extent to which the messages made participants

think of the relevant identity (overall $M = 3.52$), all $p > .28$. However, clear differences emerged for the importance that participants ascribed to the identities of environmentally concerned citizen ($M = 5.16$), hotel guest ($M = 3.84$), and guest in a particular room ($M = 2.98$), all $t(62) > 4.29$, all $p < .001$. Thus, as in the second experiment by Goldstein et al. [1], the identity linked to the provincial norm was considered the least personally important, and both the general and provincial norm identities were considered less important than that of an environmentally concerned individual.

Sample Size and Considerations of Statistical Power. To analyze the effects of the various message conditions on hotel guests' behavior, we used chi-square tests, as did Goldstein et al. [1]. We did not aim for a particular sample size, but rather collected all the data we could get within a pre-specified time period of five weeks that was agreed with the hotel. Effective sample sizes for analyses varied somewhat across dependent variables because of occasional missing values: There were 717 to 723 valid cases for the test of all normative messages combined versus the general message, which provides us with statistical power of .76 to detect a small effect ($\phi = .10$), and power greater than .99 to detect a medium-sized ($\phi = .30$) or large ($\phi = .50$) effect [22]. For testing the effects of temporal proximity and general versus provincial norms, the relevant sample sizes (which do not include the standard message condition) varied between 571 and 576, which corresponds to statistical power of .67 to detect a small effect, and power greater than .99 to detect medium-sized or large effects [22].

Results

We first analyzed overall towel reuse rates as defined by Goldstein et al. [1], counting as an instance of reuse if any used towel was placed on the towel rack on a guest's first eligible day; so each guest participated only once. Reuse rates were much higher overall (82.3%) than in the original studies (for percentages by condition, see Table 1). A planned comparison showed that the four descriptive norm conditions combined (81.9%) did not fare better than the standard environmental message (83.7%), $X^2(1, N = 723) = 0.24$, p = .62. Further planned comparisons showed that, in contrast to the original study, the same room norm conditions (78.0%) produced a significantly *lower* compliance rate than did the hotel guest norm conditions (85.6%), $X^2(1, N = 576) = 5.67$, $p = .017$, $\phi = .10$. The temporal proximity manipulation had no significant effect, with rates of 82.8% in the "current study" conditions and of 81.2% in the "completed study" conditions, $X^2(1, N = 576) = 0.27$, $p = .60$.

The specific reuse rates of hand towels and bath towels showed parallel patterns (see Table 1). The reversal of the provincial vs. general norm effect was significant for hand towels (63.3% vs. 71.3%), $X^2(1, N = 571) = 4.17$, $p = .041$, $\phi = .09$, and marginal for bath towels (65.9% vs. 72.6%), $X^2(1, N = 572) = 3.01$, $p = .083$, $\phi = .07$.

Discussion

These results suggest that towel reuse rates may be much higher overall in Germany than they are in the USA, which may reflect a higher degree of environmental awareness at the cultural level (see also [12]). Of course, the hotel studied by Goldstein et al. [1] and the hotel of the present study may differ on other (unknown) dimensions that may contribute to the difference. Importantly, the results do not support the notion that descriptive norm messages fare any better than the standard environmental message. In this respect, our findings are similar to those that Reese and his colleagues obtained in Austria and Switzerland [12]. Although one might argue that the high overall compliance rate may have obscured any differences caused by the normative messages, we nonetheless did observe one significant difference: The provincial norm relating to guests staying in the same room was significantly less effective than the general norm relating to hotel guests. This finding, while being descriptively consistent with [11], stands in contrast to an opposite effect reported in [1] and [12].

A limitation of the present study is that we do not know how guests would have behaved if there were no message at all urging them to reuse their towels. Therefore, in order to test whether the standard and normative messages would increase towel reuse rates compared to a no-intervention baseline, we repeated our study in a hotel that initially had no towel-reuse program in place.

Study 2

Method

Participants. Over a six-week period, we collected data on 204 instances of potential towel reuse in all 56 rooms of a three-star hotel located in the outskirts of the same town as in Study 1. Again, most of the observations (175) came from rooms with single occupancy; in addition, there were 27 cases of double occupancy, and two cases of three people sharing a room. Again, following [1], we used all available observations in our main analyses, but also conducted additional analyses using only instances of single occupancy.

Materials, Procedure, and Design. Materials and procedure were the same as in Study 1. The design was very similar, with the addition of a one-week, no-message baseline observation period that preceded the experimental intervention. During the following five weeks, the same five conditions as in Study 1 were

Table 1. Towel Reuse Rates (in Percent) by Message Condition (Study 1).

Dependent Variable	Message condition				
	Completed study/ Hotel guests	Completed study/ Same room	Standard environmental message	Current study/Hotel guests	Current study/Same room
	($n = 148$)	($n = 160$)	($n = 147$)	($n = 151$)	($n = 117$)
**Any towel reused**	84.5	78.1	83.7	86.8	77.8
**Hand towel reused**	70.1	61.0	68.5	72.5	66.4
**Bath towel reused**	72.1	66.7	66.2	73.2	65.0

Note. Due to occasional missing values, valid *n* per condition for hand towel reuse, from left to right, was 147, 159, 146, 149, 116, and valid *n* per condition for bath towel reuse, from left to right, was 147, 159, 148, 149, 117.
doi:10.1371/journal.pone.0104086.t001

run in such a way that each condition appeared once on each floor of the hotel.

Sample Size and Considerations of Statistical Power. Again, we did not aim for a particular sample size, but collected all the data we could get within the pre-specified time periods that were agreed with the hotel. Effective sample sizes for the main analyses varied between 132 and 204, which means that statistical power to detect small effects was insufficient (between .21 and .30), but statistical power to detect at least medium-sized effects was satisfactory (between .93 and .99) [22].

Results

The overall towel reuse rate (76.5%) was somewhat lower than in Study 1 (see Table 2 for percentages by condition). This was mainly due to the no-intervention baseline (64.3%), which differed markedly from the five message conditions combined (79.6%), $\chi^2(1, N = 204) = 4.36$, $p = .037$, $\phi = .15$. Furthermore, the standard environmental message (93.3%) was more effective than the four descriptive norm messages combined (76.5%), $\chi^2(1, N = 162) = 4.26$, $p = .039$, $\phi = .16$. Focusing on the descriptive norm conditions, in this study the provincial norm messages (82.0%) appeared to be more effective than the general norm messages (71.8%), although this difference was not significant, $\chi^2(1, N = 132) = 1.88$, $p = .17$, $\phi = .12$. The temporal proximity manipulation again had no significant effect, with compliance rates of 73.9% and 79.4% for the completed and current study conditions, respectively, $\chi^2(1, N = 132) = 0.55$, $p = .46$. Separate analyses for hand towels and bath towels again yielded comparable results (see Table 2 for percentages by condition).

As the number of people occupying a room varied more widely than in Study 1, we tested whether reuse rates would depend on the number of people in the room. On the one hand, one might expect to observe some degree of diffusion of responsibility in shared rooms, which would reduce the likelihood of each individual person complying with the reuse request; on the other hand, the a priori probability of any one person out of two (or three) reusing their towel is higher than the a priori probability of exactly one person reusing his or her towel. In line with the latter possibility, reuse rates tended to be higher in rooms shared by more than one person (89.7%) than in rooms occupied by a single person (74.3%), $\chi^2(1, N = 204) = 3.27$, $p = .071$, $\phi = .13$.

We then repeated the main analyses for the 175 single-occupancy cases, so that each data point reflected the behavior of one single individual. The results of these analyses were very similar to those including the full sample: The overall reuse rate was 74.3%. The no-intervention baseline (57.6%) was lower than the five intervention conditions combined (78.2%), $\chi^2(1, N = 175) = 5.95$, $p = .015$, $\phi = .18$. Furthermore, the standard environmental message (91.7%) tended to be more effective than the four descriptive norm messages combined (75.4%), $\chi^2(1, N = 142) = 3.08$, $p = .079$, $\phi = .15$. The provincial norm messages (81.5%) and the general norm messages (70.3%) did not differ, $\chi^2(1, N = 118) = 1.97$, $p = .16$, $\phi = .13$. The temporal proximity manipulation also had no effect (73.4% vs. 77.8% for completed vs. current study conditions, resp.), $\chi^2(1, N = 118) = 0.30$, $p = .59$. Separate analyses for hand towels and bath towels yielded comparable results.

Discussion

Despite its relatively small number of observations, Study 2 showed that presenting any message did increase towel reuse rates compared to not presenting a message. As in Study 1, the standard environmental message again was highly effective; it even surpassed the effectiveness of the descriptive norm messages. Whereas the temporal proximity manipulation made hardly any difference, there was a nonsignificant trend toward greater effectiveness of the provincial norm than the general norm. In this regard, Study 2 descriptively replicated a key finding of Goldstein et al. [1] but diverged from results of our own Study 1.

The overall reuse rates were again very high in Study 2. Analyses taking into account the number of people in a room further showed that reuse rates were even higher in rooms that were shared than in rooms with single occupancy. However, as the proportion of observations with double occupancy was rather low in both of our studies, it is unlikely that this would explain the higher overall reuse rates in our studies compared to those conducted in the USA [1], [11].

General Discussion

Despite highly similar procedures, field experiments in two German hotels yielded partly divergent findings compared to previous results that were obtained in a U.S. hotel [1]. First of all, overall reuse rates were dramatically higher in the current studies, ranging roughly between 70 and 90 percent in the message conditions, compared to the U.S. studies, where they ranged between 35 and 50 percent. Even the no-message baseline in our Study 2 was higher than reuse rates in each of the message conditions employed by Goldstein and his colleagues [1]. These figures may reflect a general difference in environment-related attitudes and behaviors between the two countries [23], [24], or

Table 2. Towel Reuse Rates (in Percent) by Message Condition (Study 2).

	Message condition					
**Dependent variable**	**No-Message Baseline**	**Completed study/ Hotel guests**	**Completed study/ Same room**	**Standard environmental message**	**Current study/Hotel guests**	**Current study/Same room**
	**(*n* = 42)**	**(*n* = 35)**	**(*n* = 34)**	**(*n* = 30)**	**(*n* = 36)**	**(*n* = 27)**
**Any towel reused**	64.3	68.6	79.4	93.3	75.0	85.2
**Hand towel reused**	47.6	65.7	67.6	90.0	72.2	81.5
**Bath towel reused**	47.6	68.6	79.4	86.7	69.4	74.1

doi:10.1371/journal.pone.0104086.t002

between the United States and central Europe more generally [12].

The higher baseline in environmental behaviors may be taken to suggest that a descriptive norm of 75% simply is not high enough to have much of an added effect over and above the standard environmental message – which indeed it did not have either in our studies or in research by Reese et al. [12]. On the other hand, our pilot participants' estimates of towel reuse rates were generally well below 75%, so we may assume that the guests participating in our experiments did not perceive the normative messages as presenting a surprisingly low figure. In a more general sense, the issue of greatly diverging baselines points to conceptual issues in trying to devise a "direct" replication: Identical operationalizations simply may take on different meanings for people in different cultures [25], [26]. So one may argue that presenting a descriptive norm of, say, 90% to German hotel clients might have constituted a closer replication of Goldstein et al. [1] than sticking to their original figure of 75% (see also [27]).

However, rather than the size of the normative majority being too small, it is also possible that European participants, being more highly involved with environmental issues, generally pay less attention to non-content cues such as descriptive norms regarding environmental behavior. Instead, they may base their behavioral decisions more strongly on the content of the issue at hand [28], [29]. This would be in line with correlational research showing that high personal involvement weakened the relationship between descriptive normative beliefs and conservation behavior [30]. It would also explain why in our Study 2, for (presumably) highly involved individuals, any message that highlighted environmental preservation worked better than no message. In a similar vein, the standard environmental message may have been more effective with German recipients because of its clear focus on environmental protection (rather than on others' behavior), which may have matched the recipients' concerns more closely.

Nonetheless, even highly involved participants often do use non-content information, but when they do, take its details into account more systematically [28], [31]. This may help to explain the reversal of the provincial norm effect in Study 1. Individuals who strongly value environmental protection and are thus more involved when processing the normative messages may be more sensitive to variations in the sample size connected with a descriptive norm. Indeed, previous research has shown that people who are highly involved in an issue take into account the sample size from which a majority norm derives, applying the "law of large numbers," and are thus more persuaded by large-sample rather than small-sample majorities [8]. However, as the direction of provincial versus general norm effects was inconsistent across studies, and as our alternative manipulation of temporal proximity showed no clear-cut results, further research seems warranted to establish the validity and limitations of the effects of more immediate versus more distal descriptive norms.

More specifically, future research on the effects of descriptive norms in applied settings would benefit from taking into account the cultural background of the people studied along with its potential implications for processing normative information, both in isolation and in conjunction with other information. As we have discussed, populations from different cultures may represent both different levels of involvement with (and background knowledge of) an issue and different a-priori probabilities of showing the target behavior. These two factors, which are related, should jointly affect the relative impact and credibility of communicated levels of social proof. Thorough pilot testing that is informed by this discussion may thus contribute to tailoring interventions aimed at changing the behavior of specific target populations.

Acknowledgments

The authors would like to thank the management and staff of the hotels where studies were conducted for their support. Many thanks also to Yasemin Coskun, Inga Hohnemann, and Laura Saldarriaga for their help in preparing and conducting the reported research, as well as to Bob Cialdini and Tina Glaser for their insightful comments on a previous draft. The authors acknowledge support for the article processing charge by the Deutsche Forschungsgemeinschaft and the Open Access Publication Funds of Bielefeld University Library.

Author Contributions

Conceived and designed the experiments: GB LES. Performed the experiments: LES. Analyzed the data: GB LES. Contributed reagents/materials/analysis tools: GB LES. Wrote the paper: GB LES. Liaised with participating hotels: GB LES.

References

1. Goldstein NJ, Cialdini RB, Griskevicius V (2008) A room with a viewpoint: Using social norms to motivate environmental conservation in hotels. Journal of Consumer Research 35: 472–482. doi:10.1086/586910
2. Cialdini RB, Kallgren CA, Reno RR (1991) A focus theory of normative conduct. Advances in Experimental Social Psychology 24: 201–234. doi:10.1016/S0065-2601(08)60330-5
3. Goldstein NJ, Cialdini RB (2009) Normative influences on consumption and conservation behaviors. In: Wänke M, editor. Social psychology of consumer behavior. New York: Psychology Press. pp. 273–296.
4. Terry DJ, Hogg MA (1996) Group norms and the attitude–behavior relationship: A role for group identification. Personality and Social Psychology Bulletin 22: 776–793. doi:10.1177/0146167296228002
5. Bohner G, Pina A, Viki GT, Siebler F (2010) Using social norms to reduce men's rape proclivity: Perceived rape myth acceptance of out-groups may be more influential than that of in-groups. Psychology, Crime & Law 16: 671–693. doi:10.1080/1068316X.2010.492349
6. Heider F (1958) The psychology of interpersonal relations. New York: Wiley.
7. Zell E, Alicke MD (2009) Contextual neglect, self-evaluation, and the frog-pond effect. Journal of Personality and Social Psychology 97: 467–482. doi:10.1037/a0015453
8. Darke PR, Chaiken S, Bohner G, Einwiller S, Erb H, et al. (1998) Accuracy motivation, consensus information, and the law of large numbers: Effects on attitude judgment in the absence of argumentation. Personality and Social Psychology Bulletin 24: 1205–1215. doi:10.1177/01461672982411007
9. Bollinger B, Gillingham K (2012) Peer effects in the diffusion of solar photovoltaic panels. Marketing Science 31: 900–912. doi:10.1287/mksc.1120.0727
10. McShane BB, Bradlow ET, Berger J (2012) Visual influence and social groups. Journal of Marketing Research 49: 854–871. doi:10.1509/jmr.11.0223
11. Schultz WP, Khazian AM, Zaleski AC (2008) Using normative social influence to promote conservation among hotel guests. Social Influence 3: 4–23. doi:10.1080/15534510701755614
12. Reese G, Loew K, Steffgen G (2014) A towel less: Social norms enhance pro-environmental behavior in hotels. The Journal of Social Psychology 154: 97–100. doi:10.1080/00224545.2013.855623
13. Rosenthal R (1990) How are we doing in soft psychology?. American Psychologist 45: 775–777. doi:10.1037/0003-066X.45.6.775
14. Makel MC, Plucker JA, Hegarty B (2012) Replications in psychology research: How often do they really occur. Perspectives on Psychological Science 7: 537–542. doi:10.1177/1745691612460688
15. Nosek BA, Spies JR, Motyl M (2012) Scientific utopia: II. Restructuring incentives and practices to promote truth over publishability. Perspectives on Psychological Science 7: 615–631. doi:10.1177/1745691612459058
16. Brandt MJ (2013) Do the disadvantaged legitimize the social system? A large-scale test of the status-legitimacy hypothesis. Journal of Personality and Social Psychology 104: 765–785. doi: 10.1037/a0031751
17. Burger JM (2009) Replicating Milgram: Would people still obey today. American Psychologist 64: 1–11. doi: 10.1037/a0010932
18. Doyen S, Klein O, Pichon C, Cleeremans A (2012) Behavioral priming: it's all in the mind, but whose mind. PLoS ONE 7 (1): e29081. doi: 10.1371/journal.pone.0029081
19. Grau I, Bohner G (2014) Anchoring revisited: The role of the comparative question. PLoS ONE 9 (1): e86056. doi: 10.1371/journal.pone.0086056
20. Barreto M, Ellemers N (2000) You can't always do what you want: Social identity and self-presentational determinants of the choice to work for a low-

status group. Personality and Social Psychology Bulletin 26: 891–906. doi:10.1177/01461672002610001

21. Garcia SM, Tor A (2009) The N-effect: More competitors, less competition. Psychological Science 20: 871–877. doi:10.1111/j.1467-9280.2009.02385.x
22. Cohen J (1988) Statistical power analysis for the behavioral sciences. Hillsdale, NJ: Lawrence Erlbaum Associates.
23. Rosenthal E (2009) What makes Europe greener than the U.S.? Available: http://e360.yale.edu/feature/what_makes_europe_greener_than_the_us/2193/. Accessed 22 January 2014
24. World Bank (2014) CO2 emissions (metric tons per capita). Available: http://data.worldbank.org/indicator/EN.ATM.CO2E.PC. Accessed 22 January 2014
25. Sedikides C, Gaertner L, Toguchi Y (2003) Pancultural self-enhancement. Journal of Personality and Social Psychology 84: 60–79. doi:10.1037/0022-3514.84.1.60
26. Stroebe W, Strack F (2014) The alleged crisis and the illusion of exact replication. Perspectives on Psychological Science 9: 59–71. doi:10.1177/1745691613514450
27. Erb H, Bohner G, Hewstone M, Werth L, Reinhard M (2006) Large minorities and small majorities: Interactive effects of inferred and explicit consensus on attitudes. Basic and Applied Social Psychology 28: 221–231. doi:10.1207/s15324834basp2803_2
28. Bohner G, Moskowitz GB, Chaiken S (1995) The interplay of heuristic and systematic processing of social information. European Review of Social Psychology 6: 33–68. doi:10.1080/14792779443000003
29. Petty RE, Cacioppo JT, Goldman R (1981) Personal involvement as a determinant of argument-based persuasion. Journal of Personality and Social Psychology 41: 847–855. doi:10.1037/0022-3514.41.5.847
30. Göckeritz S, Schultz PW, Rendón T, Cialdini RB, Goldstein NJ, et al. (2010) Descriptive normative beliefs and conservation behavior: The moderating roles of personal involvement and injunctive normative beliefs. European Journal of Social Psychology 14: 514–523. doi:10.1002/ejsp.643
31. Bohner G, Crow K, Erb H, Schwarz N (1992) Affect and persuasion: Mood effects on the processing of persuasive message content and context cues and on subsequent behaviour. European Journal of Social Psychology 22: 511–530. doi:10.1002/ejsp.2420220602
